# Awareness of reporting practices and barriers to incident reporting among nurses

**DOI:** 10.1186/s12912-023-01376-9

**Published:** 2023-07-03

**Authors:** Islam Oweidat, Khalid Al-Mugheed, Samira Ahmed Alsenany, Sally Mohammed Farghaly Abdelaliem, Majdi M. Alzoubi

**Affiliations:** 1 Nursing Administration at Zarqa University, Zarqa, Jordan; 2 College of Nursing, Riyadh Elm University, Riyadh, Saudi Arabia; 3 Department of Community Health Nursing, College of Nursing, Princess Nourah Bint Abdulrahman University, P.O. Box 84428, Riyadh, 11671 Saudi Arabia; 4 Department of Nursing Management and Education, College of Nursing, Princess Nourah Bint Abdulrahman University, P.O. Box 84428, Riyadh, 11671 Saudi Arabia; 5 Faculty of Nursing, Al-Zaytoonah University of Jordan, Amman, Jordan

**Keywords:** Awareness, Practice, Barriers, Incident reporting, Nurses

## Abstract

**Background:**

Adequate incident reporting practices for clinical incident among nurses and even all healthcare providers in clinical practice settings is crucial to enhance patient safety and improve the quality of care delivery. This study aimed to investigate the level of awareness of incident reporting practices and identify the barriers that impact incident reporting among Jordanian nurses.

**Methods:**

A descriptive design using a cross-sectional survey was employed among 308 nurses in 15 different hospitals in Jordan. Data collection was conducted between November 2019 and July 2020 using an Incident Reporting Scale.

**Results:**

The participants showed a high level of awareness of the incident reporting with a mean score of 7.3 (SD = 2.5), representing 94.8% of the highest score. Nurses perceived their reporting practices at the medium level, with a mean score of 2.23 out of 4. The main reporting barriers included worrying about disciplinary actions, fearing being blamed, and forgetting to make a report. In regard to awareness of incident reporting, there were statistically significant differences in the mean for total awareness of the incident reporting system scores according to the type of hospital (*p* < .005*). In regard to self-perceived reporting practices, nurses working in accredited hospitals demonstrated statistically significant differences in self-perceived reporting practices (t = 0.62, *p* < .005).

**Conclusions:**

The current results provide empirical results about perceived incident reporting practices and perceived barriers to reporting frequently. Recommendations are made to urge nursing policymakers and legislators to provide solutions for those barriers, such as managing staffing issues, nursing shortage, nurses’ empowerment, and fear of disciplinary actions by front-line nurse managers.

## Introduction

The persistence of clinical incident, errors, preventable adverse events, and hazards threatens patient safety and increases the burden of care, costs of care, and length of stay for patients which may lead to increased mortality of patients [1]. Indeed, more than 15% of healthcare organizations’ budget is spent on extra hospitalization costs, litigations, and other consequences of errors [2]. One in 10 hospitalized patients faces an adverse event during their hospitalization [3, 4], and nurses account for most errors, according to studies [5, 6].

As members of multidisciplinary teams, nurses play an essential role in providing a variety of care practices to patients in hospitals [7]. Nursing is considered a demanding job because it requires multitasking and poses a heavy workload, in addition to the need to provide specialized care to seriously ill and dependent patients can be intimidating for nurses [8]. Incident highly affect the safety of patients, their families, the staff, and the success of the whole organization. Incident reporting practices comprise how often nurses promptly and adequately report clinical incident, errors, preventable adverse events, and hazards [9]. However, many clinical incidents are underreported [10–12]. According to an Egyptian study, nurses need improvement in knowledge, attitude, and practices related to incident reporting [5].

Incident reporting practices of nurses and safety practices are highly related to nurses’ perceptions of their organizational culture, including values, behaviors, ways of communication, spreading myths and gossip, attitudes, and commitment to safety, which impacts the patient care quality [13, 14]. However, adequate incident reporting practices for clinical incident among nurses, and even all healthcare providers in clinical practice settings, is crucial to enhance patient safety and improve the quality of care delivery [6].

To achieve high care quality, managers need to have sufficient information on incident rates and types. Therefore, it is essential to encourage adequate reporting practices among nurses. Gathering all necessary information on patient safety reported by healthcare practitioners can assist healthcare managers in understanding system errors and create changes to decrease the probability of incident reoccurrence [6, 15]. Thus, adequate reporting for all types of incident by healthcare practitioners is paramount for patients’ safety as well as organizational success.

Several factors are associated with the under-reporting of incident reporting, such as lack of knowledge, time, workload, personal fear, and embarrassment from the manager and colleagues [15]. Addressing the correct incident reporting practices, identifying factors that contribute to the under-reporting of an incident, and assessing the preventive measures can ultimately help minimize the occurrence of incident and enhance reporting practices. In Jordan and other countries, few studies have discussed nurses’ and other health professionals’ awareness about reporting incident and evaluating reporting barriers [12, 13, 16]. Mrayyan et al. stated that during a nurse’s career, an average of 2.2 errors per a Jordanian nurse were reported with 42.1% as the rate of reported medication errors [12]. The aims of this study were to investigate the level of awareness of incident reporting practices and identify the barriers that impact incident reporting among Jordanian nurses, followed with research questions:

1. What is the nurse’s level of awareness of incident reporting?
2. What is the nurse’s level of self-perceived of incident reporting practices?
3. Is there any difference between awareness and self-perceived of incident reporting practices and nurse’s socio-demographic?

## Methods

### Research design, sample, and sampling

A descriptive design was employed. A sample of 382 nurses from 15 different hospitals was included by multistage sampling. The hospitals were selected via a simple random sampling technique from the middle, north, and east governate to enhance the representativeness of all regions in Jordan. Each region was represented in the hospitals’ sample by selecting the number of hospitals according to the total number of hospitals. The selection of hospital type (i.e., governmental, or private) from each region was made according to the percentage of each type in each region. Hospitals with a capacity of fewer than 60 beds were excluded because of the small number of registered nurses on their duty schedules, and they tended to be peripheral hospitals as well.

In the second sampling stage, nurses were selected via a nonprobability convenience sample of those working as registered nurses in the selected hospitals. Nurses were included if they had at least a Bachelor’s degree in nursing with at least one year of nursing experience. Nurses working in outpatient or other non-practice areas were excluded. In addition, nurse managers or others in administrative positions or those not in direct patient care were also excluded. The sample size was estimated using G power (version 3.1.5) for one-way ANOVA, 95% power, a medium effect size of 0.25, and an alpha level of ≤ 0.05. According to this formula, the minimum required sample size was 319 participants. The population was oversampled to account for a possible attrition rate of participants. However, 325 nurses were included in the initial sample, and the final sample included 308 nurses.

### Measurement

Socio-demographic variables included age, gender, level of education, years of experience in nursing, hospital type, type of hospitals ward or unit, and if the hospital was accredited (e.g., Joint Commission International, Health Care Accreditation Council) or not. Evans et al. developed a modified version of the Incident Reporting Scale comprising three sections [17]. The first section measures awareness of the incident reporting system with five yes or no questions related to awareness of the incident reporting system. To calculate mean awareness, the yes answer was scored as 2 and the wrong answer was scored as 1, with a total score ranging from 5 to 10. Where the higher score represents higher awareness.

The second section includes self-perceived reporting practices in which participants respond to 11 items representing a diverse range of incident. Participants’ responses can be made on 1- 4 point Likert scale (never, < 50% of occasions, 50% or more of occasions, always). The total self-perceived reporting practices score was classified as ‘low level’ (< 50th percentile), ‘medium level’ (50th and 75th percentiles), and ‘high level’ (> 75th percentile). Additionally, participants are asked to comment on how often they do (actual reporting practices) and how often they think they should report (their views on the necessity of reporting). The third section includes 19 items to address self-perceived barriers to reporting incident. In this section, participants are asked to report their degree of agreement on a 5-point Likert scale where 1 = strongly agree, and 5 = strongly disagree.

Content validity was assessed in previous studies through a panel of experts using the Q-sort technique to classify themes among data. There was an agreement on categorizing questions related to reporting barriers assuring its content validity (Kappa = 0.7). Also, there was moderate agreement on categorizing questions related to types of incident (Kappa = 0.6). Additionally, test–retest reliability was done using Kappa ≥ 0.4. The Cronbach’s alpha for the scale of frequency of incident reporting in the current study was 0.97.

### Data collection procedures

Data collection was conducted between November 2019 and July 2020. The researchers obtained a list of nurses and their working schedules to arrange a time for data collection at their convenience. Nurses were screened for their eligibility to participate in the current study; then, each nurse was asked to fill in the questionnaire, seal it in the attached envelope, and put it in the box placed at the nursing counter at each department.

### Data analysis 

The Statistical Package for Social Science (SPSS version 23) for Windows was used for data analysis (IBM, 2019). Descriptive statistics (frequency, percentages, and mean) were computed for the demographic. Parametric tests (mean SD, independent sample t-tests, and ANOVA) were performed on normally distributed data to examine awareness of the incident reporting and perceived reporting practices and associations between categorical variables. The chosen level of significance is *p* < 0.05.

## Results

### Demographic characteristics of participants

A total of 308 nurses completed the study with a response rate of 89%. More than half nurses were female (56%), with a mean age of 29.7 ± 5.11, ranging from 23–50 years old, with 7 years of experience in average. One hundred thirty-eight nurses were recruited from private hospitals, 90 from governmental hospitals, and 80 from university-affiliated hospitals, in addition, 86.8% had a Bachelor’s degree. Accredited hospitals constituted 67.7% of all hospitals (Table 1).

**Table 1:** Demographic characteristics of participants (*N* = 308)

Variables	Frequency	Percentage (%)
**Age 29.7(5.11) M (SD)**		
**Gender**		
Male	133	44
Female	175	56
**Type of hospital**		
Governmental	90	30.2
Private	138	43.6
University affiliated	80	26.2
**Level of nursing education**		
Bachelor	265	86.8
Postgraduate	43	13.1
**Years of experience in nursing** 6.9 ± 5.0		
1–5 Year	149	49.2
6–10 Year	119	38.8
11 Year above	40	12.0
**Type of hospitals ward or unit**		
Medical /Surgical	96	29.5
Emergency Room	28	8.6
Critical care unit	94	28.9
Pediatric	34	10.5
Psychiatric	3	0.9
Operation room	12	3.7
Gynecology	24	7.4
Others	34	10.5
**The hospital Accreditation**		
Accredited (HCAC/JCI)	220	67.7
Not accredited	105	32.3

The participants showed a high level of awareness of the incident reporting with a mean score of 7.3 (SD = 2.5), representing 94.8% of the highest score. Most participants were aware of the existence of an incident reporting system (94.8%). Two-thirds of them have previously filled out incident forms and knew about access to them. One-third of participants filled out the incident in the last month (32.6%) (Table 2).

**Table 2:** Awareness of the incident reporting (*N* = 308)

Items	Frequency (%)
**Existence of incidents reporting system**	
Yes	291 (94.8)
No	17 (5.2)
**Filling in an Incident Form**	
Yes	196 (60.3)
No	115 (39.7)
**Knowing how to access the incident form**	
Yes	225 (69.3)
No	83 (30.7)
**Filling in an Incident in the Last Month**	
Yes	106 (32.6)
No	202 (67.4)
**Knowing What to do with the Completed Incident Form**	
Yes	207 (63.7)
No	101 (36.3)

The results revealed that registered nurses perceived their reporting practices at the medium level, with a mean score of 2.23 out of 4. which represents 66.0% of the highest possible score.???

Only (24.8%) always reported incidents of pressure sore for their patient, whereas more than half of participant’s think that they should report the incidents (53.4%). One quarter of participants (20.8%) always reported of DVT post-operatively incidents due to inadequate prophylaxis, whereas less than half think that they should report the incidents (48.7%) (Table 3).

**Table 3:** Self-perceived reporting practices (*N* = 308)

Question			Never %	Less than 50% of occasions	50% or more of occasions	Always %
1	Patient sustained a pressure sore whilst in hospital	How often do you report this incident?	26.2	33.7	15.3	24.8
		How often do you think you should report it?	13.2	16.2	17.2	53.4
2	Patient sustained an injury due to a fall in hospital	How often do you report this incident?	26.2	32.8	17.4	23.6
		How often do you think you should report it?	10.8	15.3	23.8	50.1
3	Patient sustained a hospital-acquired infection, e.g., infected wound site, phlebitis due to infected IV site	How often do you report this incident?	31.0	31.1	18.2	19.7
		How often do you think you should report it?	15.3	17.5	22.8	44.4
4	Patient sustained a DVT post-operatively due to inadequate prophylaxis	How often do you report this incident?	35.8	27.7	15.7	20.8
		How often do you think you should report it?	13.5	15.5	22.2	48.7
5	Patient received the wrong treatment or procedure	How often do you report this incident?	28.8	26.8	16.2	26.2
		How often do you think you should report it?	14.2	13.2	20.0	52.5
6	Patient did not receive the necessary treatment or procedure	How often do you report this incident?	30.8	23.7	22.2	23.3
		How often do you think you should report it?	13.2	17.1	20.9	48.8
7	Staff made a drug error, but it was not actually given (near miss)	How often do you report this incident?	26.5	22.5	23.7	22.2
		How often do you think you should report it?	15.1	13.8	19.3	51.8
8	Staff made a drug error where no corrective treatment was necessary	How often do you report this incident?	26.5	22.5	22.2	23.7
		How often do you think you should report it?	13.5	13.7	21.9	50.7
9	Staff made a drug error resulting in correcting treatment being given	How often do you report this incident?	27.9	28.8	19.1	24.2
		How often do you think you should report it?	17.4	13.2	23.5	45.9
10	Problem with equipment or machinery resulting in patient harm, e.g., Faulty pump/ bed	How often do you report this incident?	28.2	30.0	23.1	18.7
		How often do you think you should report it?	15.5	15.9	22.8	45.8
11	Breach in confidentiality, e.g., Information given without authority	How often do you report this incident?	31.2	30.3	22.7	14.8
		How often do you think you should report it?	13.8	15.1	31.5	39.6

In this study, about half of the participants were worried about disciplinary actions (51.3%). The participants feared being blamed mostly by junior staff (46.5%). Less than half (43.5%) did not want to get into trouble. Also, 41.0% of the participants forgot to make a report. Few participants strongly agree that they will not get feedback of report something (11.6%). Around a quarter of the participants (20.2%) neither not responsible to report somebody else’s mistakes. Less participants (10.2%) strongly agree that their co-workers may be unsupportive. See Table 4.

**Table 4:** Reporting barriers as perceived by nurses (*N* = 308)

NO	Barrier to Reporting	Strongly Disagree	Disagree	Neither	Agree	Strongly Agree
		**N (%)**	**N (%)**	**N (%)**	**N (%)**	**N (%)**
1.	I am worried about disciplinary action	30 (9.2%)	38 (12.7)	57 (18.5)	156 (51.3)	27 (8.3)
2.	I don’t want to get into trouble	23 (7.1)	64 (20.7)	60 (19.7)	138 (43.5)	23 (7.1)
3.	If I report something, I never get any feedback on what action is taken	16 (4.9)	74 (23.8)	72 (23.2)	109 (36.5)	37 (11.6)
4.	I feel that if I discuss the case with the person involved, nothing else needs to be done	24 (7.4)	69( 21.4)	66 ( 20.3)	115 (40.4)	34 (10.5)
5.	I worry about who else is privy to the information that I disclose	20 (6.2)	61 (18.8)	77 (23.7)	120 (36.9)	30 (9.2)
6.	It’s not my responsibility to report somebody else’s mistakes	30 (9.2)	71 (21.8)	65 (20.2)	115 (40.4)	27 (8.5)
7.	My co-workers may be unsupportive	25 (7.7)	67 (20.8)	81 (24.9)	102 (36.4)	33 (10.2)
8.	I am worried about litigation	20 (6.2)	64 (20.7)	80 (25.6)	112 (39.5)	26 (8.0)
9.	Even if I don’t give my details, I’m sure that they’ll track me down	20 (6.2)	63 (19.4)	92 (28.5)	107 (37.9)	26 (8.0)
10.	When the ward is busy, I forget to make a report	22 (6.8)	58 (17.8)	70 (21.8)	117 (41.0)	41 (12.6)
11.	I don’t feel confident that the form is kept anonymous	22 (6.8)	59 (18.2)	92 (28.5)	108 (37.0)	31 (9.5)
12.	The incident form takes too long to fill out, and I just don’t have time	23 (7.1)	63 (19.8)	87 (26.8)	109 (37.5)	27 (8.8)
13.	The incident was too trivial	16 (4.8)	65 (20.0)	80 (24.0)	116 (41.0)	36 (10.2)
14.	Junior staff are often blamed unfairly for adverse incident	24 (7.4)	64 (20.7)	65 (20.2)	138 (46.5)	17 (5.2)
15.	When the incident does not eventuate or a correction is made (a near miss), then I don’t see any point in reporting it	19 (5.9)	56 (17.4)	82 (26.2)	126 (42.8)	25 (7.7)
16.	Adverse incident reporting is unlikely to lead to system changes that will improve the quality of care	17 (5.4)	58 (17.9)	82 (26.2)	126 (42.8)	25 (7.7)
17.	The hospital form is too complicated and requires too many details	25 (7.7)	52( 16.3)	96 (31.5)	105 (35.3)	30 (9.2)
18.	I don’t want the case discussed in meetings	21 (6.7)	54 (16.8)	82 (26.2)	125 (42.1)	26 (8.2)
19.	I don’t know whose responsibility is to make a report	23 (7.1)	54 (16.8)	89 (27.4)	107 (37.9)	35 (10.8)

In regard to awareness of incident reporting, there were statistically significant differences in the mean for total awareness of the incident reporting system scores according to the type of hospital (*p* < 0.005*). Regarding gender, female participants showed higher awareness than male participants, however, no statistical significance resulted in the analysis. The participants with postgraduate degrees and having 11 years of experience or more showed higher awareness than other groups. The participants who worked in accredited hospitals showed higher awareness than those from non-accredited hospitals. See Table 5.

**Table 5:** Comparison of the participants’ awareness of the incident reporting system with their demographic

**Descriptive Characteristics**	Mean (SD)	t/F	*P* value
**Gender**		-0.031	0.37
Female	6.6 (3.2)		
Male	5.2 (1.8)		
**Type of hospital**		2.35	.005*
Governmental	5.5 (2.8)		
Private	6.7 (3.2)		
University affiliated	4.9 (2.6)		
**Level of nursing education**		2. 32	0.011
Bachelor	7.1 (4.1)		
Postgraduate	7.5 (2.8)		
**Years of experience in nursing**		-2.29	0.026
1–5 Year	5.1 (1.6)		
6–10 Year	6.7 (2.3)		
11 Year above	7.2 (4.3)		
**Hospital Accreditation**		0.426	0.020
Accredited	6.9 (3.9)		
Not accredited	5.2 (2.8)		

^*^ p value

In regard to self-perceived reporting practices, nurses working in accredited hospitals demonstrated statistically significant differences in self-perceived reporting practices (*t* = 0.62, *p* < 0.005). Regarding gender, female participants showed higher scores compared to male participants and no statistically significant differences (*t* = 341, *p* = 0.019). The participants working in private hospitals showed higher scores than other groups (*f* = 4.3, *p* = 0.022). The participants who had postgraduate degrees showed higher self-perceived reporting practices than other groups. The participants with 6–10 years of experience in nursing showed higher scores than other groups (*f* = 1.98, *p* = 0.011). See Table 6.

**Table 6:** Comparison of the participants’ self-perceived reporting practices with their demographic

**Descriptive Characteristics**	Mean (SD)	t/F	*P* value
**Gender**		34.1	0.019
Female	8.1 (3.3)		
Male	6.2 (2.7)		
**Type of hospital**		4.31	0.022
Governmental	5.5 (2.8)		
Private	6.7 (4.2)		
University affiliated	5.2 (2.4)		
**Level of nursing education**		2.76	0.015
Bachelor	6.1 (3.4)		
Postgraduate	7.5 (3.8)		
**Years of experience in nursing**		1.98	0.011
1–5 Year	6.2 (4.2)		
6–10 Year	7.1 (5.8)		
11 Year above	6.7 (4.6)		
**Hospital Accreditation**		0.62	.005*
Accredited	9.6 (5.5)		
Not accredited	6.2 (4.2)		

^*^ p value

## Discussion

The current study results showed that registered nurses had high level of awareness of incident reporting. These results were consistent with Chen et al., who found that the nurses’ perceptions toward incident reporting practices were high [18]. In addition, the vast majority of the registered nurses participating in the current study (94.8%) declared that they were aware of the existence of incident reporting systems in their healthcare institutions. These results align with the results of AbuAlRub et al., which revealed that almost all the surveyed nurses were aware that their healthcare institutions had an incident reporting system [19]. These results could be due to the efforts and activities of the accrediting bodies and the awareness campaigns held in Jordanian hospitals to increase healthcare providers’ awareness about the incident reporting system.

The results of the current study revealed that around 60% of the surveyed participants had filled in an incident report at least once in their practice, which is consistent with the results of Farzi, et al., who reported that around two-thirds of staff nurses had ever filled in an incident report [20]. However, the results of the current study are to some extent consistent with the results of Agegnehu et al., which found that 80% of the surveyed healthcare professionals had ever filled an incident report [21].

Although most participants had a high level of awareness of the incident reporting, only 32% of the surveyed nurses had filled in an incident report in the last month of the time of data collection. This result is slightly different from the findings of AbuAlRub et al. in which they found that 42.2% of the surveyed nurses had filled in an incident report in the last month at the time of data collection [19]. Many factors could have impacted this result, such as a low volume of near misses or adverse events at that time because it concerns a limited period which is one month.

The participants in the current study knew that the incident reporting system existed, but there was some uncertainty regarding how to locate or access the form, as just 69.3% of the participants knew how to locate or access the incident form. The current results align with Evans et al., which revealed that around two-thirds of the surveyed participants knew how to locate or access the incident form once needed [17]. Moreover, these results align with Alboliteeh and Almughim, who found that nearly 62% of the participants reported no confusion regarding access to the occurrence variance report (OVR) System [22]. However, the current results are considered to some extent consistent with AbuAlRub et al., in which they found that 80.8% of the surveyed nurses had reported that they knew how to locate or access an incident form in their hospitals [19]. These differences might be attributed to the inattention of some nurses to the general orientation programs at the beginning of their practical lives, wherein the incident reporting system and safety issues are discussed thoroughly [22].

Regarding the question of what to do with the completed incident form 63.7% of the participants knew what to do with the completed incident report once it was done. This result is consistent with Agalu et al., which showed that nearly two-thirds of the surveyed participants knew what to do after completing the incident form [23]. On the other hand, these results are in line with AbuAlRub et al., who found that nearly 70% of the surveyed nurses knew what to do with the completed incident form [19]. However, in Alboliteeh and Almughim’s study, almost all the surveyed participants (94.3%) had good knowledge about what to do with the completed incident report [24]. This incongruence might be because some nurses believe in-charge nurses and safety managers are responsible for proceeding with a completed incident form.

The results of the current study revealed that registered nurses perceived their reporting practices at the medium level, with a mean score of 2.23 out of 4. The results of the current study are consistent with the results of Kusumawati et al., in which they found that nurses’ perceptions toward incident reporting practices in Indonesian hospitals were above the average [25]. Many factors influence the behaviors of nurses regarding incident reporting. These factors include the clarity of reporting system, the existence of patient safety culture, workload such as staffing-related problems and heavy assignments, severity of the incident or error [26–28], and colleague support among the different units and floors [29]. Differences also might be attributed to the difference in the perception of the importance of incident reporting for quality healthcare among health professionals in different countries.

Concerning the incident that participants in the current study reported, only 26.2% of the registered nurses always reported an incident of “patient received wrong treatment or procedure.” This was the highest percentage among all incident, meaning that nurses perceived an incident of wrong treatment or procedure as important always to report. This result is consistent with the results which revealed that the wrong procedure or wrong treatment was among the top most frequently reported incident among health workers [30]. Additionally, this result was consistent with Fathi et al., who found that the wrong treatment or medication time was among the most reported incident among the surveyed nurses [31]. In contrast, the current results are paradoxical to the results conducted at Jordanian hospitals, which revealed that the surveyed nurses most often reported equipment fault resulting in patient harm, not for any other incident type [19].

Incongruencies in general might be because variables such as organizational culture, climate, perceived severity of the incident, gender of the victim, type of the ward, and perceived consequences of reporting in different healthcare systems create differences. It also might be attributed to differences in the perception of the importance of incident reporting for quality healthcare among nurses [32]. In addition, previous research may have used different instruments, study designs, or study settings than the current study which can affect the interpretations of the findings.

The current results revealed that only 14.8% of registered nurses reported an incident of “breach in confidentiality such as information given without authority” all the time, which is the lowest percentage among all incident. These current results are consistent with the results of Sakuma, who found that student nurses did not always report a breach of confidentiality of patients [33]. Wondmieneh, et al. stated that the unit type affects confidentiality reporting issues among nurses [32]. This congruence might be because some healthcare workers believe that confidentiality of patient information is not a severe event that can affect a patient’s health status and safety.

The current study showed that more than a quarter of participant’s nerve reported incident for patient sustained a DVT post-operatively due to inadequate prophylaxis. This result contradicts AbuAlRub et al. in which around 24.9% of the surveyed nurses reported “post-operative DVT due to inadequate prophylaxis [19]. Also, the recent studies nurses reported inadequate prophylaxis of DVT [34–36].

The current study revealed that 53.2% of the registered nurses did think that they should report an incident of “patient sustained a pressure sore whilst in hospital” all the time, which is the highest percentage among all incident. This result aligns with retrospective review study which showed the high incidence of pressure ulcer which might be related under-reported [37]. In another retrospective study, demonstrates that staff nurses often perform poorly on documenting pressure ulcer appearance [38, 39]. Half of the participant’s think that they report the injury due to a fall in hospital. This result does not align with Jordanian study, which revealed that 80% of the surveyed nurses thought that they should report an incident of “patient injury due to fall” all the times, which was the highest percentage [19]. The current results are highly congruent with the results of Heslop and Lu, that pressure ulcers and falls were the two most frequent outcome measures that are nursing-sensitive indicators [40].

The results of the current study revealed that 39.4% of the nurses thought that they should report an incident of “breach in confidentiality such as given information without authority” all the times, which is the lowest percentage among all incident. The current results might be explained by the fact that bedside nurses believe that pressure ulcers incident should be reported all the times (regardless its degree) because it is a frequent and common issue in all clinical settings all over the world that is given higher priority in hospital education programs than confidentiality problems. Plus, many nurses perceive a pressure ulcer incident as an indicator of poor quality of care rather than a system failure, which requires the nurses to make quick decision-making [41, 42]. Moreover, this result is slightly consistent with the results of AbuAlRub et al., in which they found that around 45% of the surveyed nurses believed that they should report an incident of “breach in confidentiality” at all times [19].

Regarding barriers of reporting that registered nurses conveyed in the current study, the results revealed that the highest frequency among all barriers was for worrying about disciplinary actions. This result aligns with study in Saudi Arabia, where “Nursing administrative response to the error” had the highest frequency among all barriers to incident reporting [43]. Taylor et al., claimed that this barrier can be reduced by the implementation of an anonymous reporting system [44].

The participants feared being blamed mostly by junior staff (46.5%). These studies also ascertain that blamed is a major barrier [45, 46], the current results could be because human beings generally do not prefer to be punished by their supervisors and managers and do not want to be fired or have their careers derailed. Few participants strongly agree that they will not get feedback of report something (11.6%), this result was agreed with recent study [47]. This negative tendency can seriously affect and distort nurses’ sense of accountability and moral obligation in the future. Therefore, active communication between nurses and the supervisors contribute for quality and assuring patients’ safety [3, 8].

Nurses working in accredited hospitals demonstrated showed higher awareness of incident reporting and self-perceived reporting practices than non-working in non-accredited hospitals. This result was similar with a systematic review that nurses in the accredited hospitals found positive safety culture, patient satisfaction and experience, and employee satisfaction [48]. However, awareness campaigns, leadership support, and better design of accreditation standards and processes are vital remedies to consider [49].

The participants with postgraduate degrees and having 11 years of experience or more showed higher awareness than other groups, the results agreed with study conducted among Jordanian nurses [50]. Studies described that when experience and education increases, hospital quality of service also increase [14, 26]. Possibly inexperienced and newly graduated healthcare givers suffer from stress regarding practice, which makes them vulnerable to an increased incidence of errors [39]. Huge numbers of staff with inadequate experience and insufficient concerns for incident reporting are a risk to patient care.

### Limitations

The study has limitations such as study design and setting being conducted not in all geographic Jordan area, so it had a problem and difficulties with generalization. Furthermore, the study used nonprobability convenience sampling, which may lead to selection bias that impairs and threatens internal validity. Also, technical difficulties were faced by some nurses were regarding electronic survey.

### Nursing implications

This study might be considered the base for further studies that will be conducted to investigate awareness of reporting practices and barriers to incident reporting on nurses in Jordan. It is recommended to conduct qualitative studies to explore in depth the barriers faced by Jordanian nurses and to understand the organizational factors and personal characteristics that may help nurses to cope with such barriers. The findings of this study indicate the importance of articulating policies and strategies that manage incident reporting in the workplace.

## Conclusion

Variations exist in the perceived barriers that hinder nurses from reporting incident due to several factors such as differences in perceptions of the barriers, personal factors such as seniority and experience, organizational culture, and work circumstances. The current results provide empirical results about perceived incident reporting practices and perceived barriers to reporting frequently, which urge nursing policymakers and legislators to provide solutions for these barriers, such as staffing issues, nursing shortage, nurses’ empowerment, and fear of disciplinary actions by front-line nurse managers.

## Data Availability

The datasets used and/or analysed during the current study available from the corresponding author on reasonable request.
